# Host adapted intramammary infections in pregnant heifers which were co-housed and reared on fresh milk as calves

**DOI:** 10.1186/1746-6148-9-49

**Published:** 2013-03-15

**Authors:** Inge-Marié Petzer, Joanne Karzis, Maia Lesosky, Johanna C Watermeyer, Renette Badenhorst

**Affiliations:** 1 Department of Production Animal Studies, Section Udder Health, Faculty of Veterinary Science, University of Pretoria, Private Bag X04, Onderstepoort, 0110, South Africa; 2 Department of Medicine, University of Cape Town, Observatory, Cape Town, 7925, South Africa

**Keywords:** Heifer intramammary infections, *Staphylococcus aureus*, *Streptococcus agalactiae*, Co-housed calves, Fresh milk rearing systems

## Abstract

**Background:**

Heifers can calve down with intramammary infections (IMI) and udder damage. This will have a negative impact on their longevity, future milk yield and financial return. Co-housed pre-weaned calves that are fed fresh milk have the opportunity to suckle each other’s teats and may infect udders of fellow heifer calves with pathogens present in milk. The prevalence of IMI in pregnant heifers in South Africa (SA) which were co-housed and reared on fresh milk as calves, is not known. Quarter secretion samples from both pregnant heifers (n = 2065) and dry cows (n = 5365) were collected for microbiological analysis from eight SA dairy herds. All heifers tested in this study were co-housed pre-weaning and were fed fresh milk as calves.

**Results:**

The prevalence of coagulase negative staphylococci, *Staphylococcus aureus*, *Streptococcus agalactiae*, environmental streptococci, coliforms and samples with no bacterial growth in heifers was 26%, 0.9%, 0.08%, 1.4%, 0.4% and 66%, respectively. The overall prevalence ratio between heifers and cows for *Staphylococcus aureus* IMI was 0.76 (95% CI: 0.59, 0.98). Four of the individual herds had prevalence ratios of less than one (p < 0.05), one herd had a prevalence ratio of 3.15 (95% CI: 1.52, 6.32), and the remaining 3 herds had a prevalence ratio not significantly different from 1.0. Marginally significant differences were found between *Staphylococcus aureus* IMI in pregnant heifers compared to cows in their second and later lactations (p = 0.06, p = 0.05, respectively) but no significant differences between heifers and cows in their first lactation.

**Conclusions:**

The presence of *Streptococcus agalactiae* IMI in heifers came as a surprise, especially as herd infection rates were low. The high prevalence ratio of *Staphylococcus aureus* between heifers and cows in one herd warrants further investigation due to the potential danger of udder damage in a young cow at the start of her productive life. The IMI in heifers with host adapted pathogens can also act as a source of new IMI for lactating dairy cows.

## Background

Mastitis has been identified as the most costly disease of dairy cows, resulting in considerable reduction in profitability for the dairy producer and industry (5^th^ International Dairy Federation (IDF) Mastitis Congress 2010, Christchurch, New Zealand). Historically, control of mastitis has been driven primarily by economic considerations. However, a growing consumer focus and demand for wholesome and safe milk and pressure through international supply chains are currently further increasing the need for quality milk [1,2].

Once the udder parenchyme is chronically infected, very little can be done to reverse the process and the productivity of that animal is crippled. When intramammary infection (IMI) occurs in a young cow or heifer, her productive life is potentially shortened which prevents her from reaching the ideal target of six lactations set by the IDF. Economic losses as a result of mastitis may actually be higher in heifers than in cows due to the extensive damage done by pathogens to the developing secretory tissue.

A New Zealand study has shown that mastitis in heifers reduces milk production in the first and each of the following lactations by 8% [3]. Intramammary infections in heifers can cause long-term elevation in somatic cell counts [4] and have a negative influence on their longevity and on the total herd economy [5].

Intramammary infections in unbred and pregnant heifers were thought to occur infrequently. During the last two decades, however IMI in heifers have clearly been shown to be present [6-10]. This is becoming a growing concern, which needs to be addressed in a pro-active udder health approach. It was shown by [11] that IMI can persist for extended periods of time in the udder of heifers and can impair development of that mammary gland. The main group of organisms isolated from IMI in heifers is the coagulase negative staphylococci (CNS). The large environmental population of CNS may contribute to the high numbers of CNS found in udders of pregnant heifers. A route of entry for bacteria is created when pressure in the udder increases close to calving and the teat canal is forced open [12].

*Staphylococcus aureus* and *Streptococcus agalactiae* are host adapted micro-organisms frequently isolated from milk samples of adult dairy cows in South Africa. These bacteria are transmitted from one udder to the next during milking by the hands of milkers, teat liners and the use of communal cloths. They are not known to survive for extended periods in the environment and are therefore unlikely to infect heifers. *Staphylococcus aureus* is however, isolated from udder secretions of heifers and the origin of this IMI needs to be determined. However, factors associated with mastitis in heifers are not completely understood. Isolating *Streptococcus agalactiae,* a known obligatory udder pathogen, from the udder secretion of a pregnant heifer is very unusual [13,14].

It is common practice in modern milk production to remove calves from their dams shortly after birth and to rear them on milk or milk replacers. Calves fed on cow’s milk achieve on average better daily weight gains, but it has a cost implication. Although not recommended, some dairy farms still feed milk from mastitis cows to calves.

Where seasonal calving is practiced, farmers often co-house calves in groups rather than in individual pens during the pre-weaning period. Housing has been shown to play an important role in the health of pre-weaned calves [6,7,9,15]. Co-housing pre-weaned calves particularly has been identified as a high risk factor for calf health due to an increased tendency to suckle the ears or teats of fellow calves [10]. When milk which contains pathogens is consumed by calves, and they suckle on teats of a fellow heifer calf, the pathogens may enter the delicate teat canal of the pre-weaned calf and remain in the udder tissue of that animal [13].

The aim of this study was to determine the prevalence of bacteria responsible for IMI in pregnant heifers in pasture based herds and compare it to that of dry cows of various parities within the same herds. Secondly, the prevalence of IMI in pregnant heifers by *Staphylococcus aureus* and *Streptococcus agalactiae*, when co-housed as pre-weaned calves and fed on fresh cow’s milk in herds positive for *Staphylococcus aureus* or *Streptococcus agalactiae*, needed to be determined.

## Results

### General microbiological results

In total 5365 cows and 2065 late pregnant heifers were enrolled in the study from 8 farms. The median number of cows per farm and interquartile range (IQR) was 651 (564, 712) and the median (IQR) number of heifers per farm was 257 (190, 322). Microbiological results obtained from udder secretions of pregnant heifers are summarized in Table 1 by herd. The overall prevalence ratio in all heifers between host adapted (*Staphylococcus aureus* and *Streptococcus agalactiae*) (n = 85) and environmental pathogens (n = 2687) is 1:30. The corresponding ratio for cows with one or more lactations is 1:12 (data not shown).

**Table 1:** Prevalence of bacteria in udder quarter secretions of heifers

**Herds (quarters)**	**No Growth**	STA*	SAG*	CNS**	STR**	ENT**	Other bact. **
	**N (%)**	**N (%)**	**N (%)**	**N (%)**	**N (%)**	**N (%)**	**N (%)**
A (1246)	844 (67.74)	6 (0.48)	0	307 (24.64)	9 (0.72)	6 ( 0.48)	74 (5.94)
B (1404)	883 (62.89	10 (0.71)	3 (0.21)	437 (31.13)	23 (1.64)	5 ( 0.36)	43 (3.06)
C (804)	576 (71.64)	0	1 (0.12)	166 (20.65)	30 (3.73)	5 (0.62)	26 (3.23)
D (1756)	1233 (70.22)	37 (2.11)	0	390 (22.21)	8 (0.46)	7 (0.4)	81 (4.61)
E (640)	363 (56.72)	14 (2.19)	0	217 (33.91)	6 (0.94)	2 (0.31)	38 (5.94)
F (1123)	883 (78.63)	4 (0.36)	3 (0.27)	178 (15.85)	22 (1.96)	6 (0.53)	27 (2.4)
G (932)	478 (51.29)	6 (0.64)	0	366 (39.27)	12 (1.29)	2 (0.21)	68 (7.3)
H (352)	225 (63.92)	1 (0.28)	0	98 (27.84)	5 (1.42)	1 (0.28)	22 (6.25)
All (8257)	5485 (66.43)	78 (0.94)	7 (0.08)	2159 (26.15)	115 (1.39)	34 (0.41)	379 (4.59)

STA = *Staphylococcus aureus*; CNS = coagulase negative staphylococci; SAG = *Streptococcus agalactiae*; STR = *Streptococcus uberis, Streptococcus dysgalactiae, Enterococcus faecalis*; ENT = Gram negative bacteria; Other bact. = *Staphylococcus pseudintermedius, Micrococcus, Bacillus* spp., & mixed growth.

* = Host-adapted pathogens. **Environmental pathogens.

### *Staphylococcus aureus* isolated from heifers and cows

Table 2 presents the detailed results for *Staphylococcus aureus* in both heifers and cows. The overall prevalence ratio (95% CI) between heifers and cows is 0.76 (0.59, 0.98) while the herd level prevalence ratio varied from a low of approximately 0 (0, 0.8) to a high of 3.17 (1.52, 6.32). *Staphylococcus aureus* was isolated in heifers from 0.94% of quarters and 3.1% udders and from 1.24% of quarters and 4.47% udders of cows (data for cows not shown). Of the 64 heifers with *Staphylococcus aureus* isolated from the udder, the majority 85.9% (n = 55) was present in a single quarter, 7.6% (n = 5) in two, 4.7% (n = 3) in three and just one individual with isolates present in all four quarters. In cows 240 udders were positive for *Staphylococcus aureus*, with 90.4% (n = 217) isolated from one quarter, 8.3% (n = 20) from two and the remaining 1.3% (n = 3) from three quarters per udder. There were no cases of a cow having positive isolates from all four quarters. Pairwise comparisons of lactation classes (pooled over all farms) and *Staphylococcus aureus* positivity at the quarter level indicated no significant difference between heifers and 1st lactation cows (p = 0.19) but approached statistically significant differences between both heifer and 2nd lactation (p = 0.06) and between heifer and 2nd lactation plus (p = 0.05). Given the unusual prevalence ratio of Farm E compared to the rest of the farms (Table 2), this same test was applied to a reduced set excluding all results from Farm E. In this case all three comparisons were statistically significant, with p-values of 0.03, 0.02 and 0.002, for heifers compared to 1st, 2nd and 2nd plus lactation cows respectively.

**Table 2:** ***Staphylococcus aureus *prevalence in udder quarter secretions of heifers and cows by parity status**

**Herd**	**Total N of quarters heifers/cows**	**STA Heifers N (%)**	**STA Cows N (%)**				**Prevalence ratio**	**95% Confidence interval**
			**First lactation**	**Second lactation**	**Second lactation plus**	**All lactation cows**		
A	1246/1882	6 (0.48)	5 (0.41)	1 (0.33)	3 (0.83)	9 (0.48)	1.01	(0.36, 2.82)
B	1404/4008	10 (0.71)	12 (1.18)	12 (1.6)	33 (1.47)	57 (1.42)	0.50	(0.26, 0.98)
C	804/2548	0 (0)	9 (1.29)	9 (1.7)	14 (1.06)	32 (1.26)	0.00	(0, 0.8)
D	1756/3243	37 (2.11)	28 (2.63)	23 (1.85)	32 (3.42)	83 (2.56)	0.82	(0.56, 1.21)
E	640/2320	14 (2.19)	1 (0.3)	4 (1.1)	11 (0.68)	16 (0.69)	3.17	(1.52, 6.32)
F	1123/2716	4 (0.36)	6 (0.9)	5 (0.87)	18 (1.22)	29 (1.07)	0.33	(0.12, 0.95)
G	932/2659	6 (0.64)	10 (1.1)	6 (1.04)	11 (0.94)	27 (1.02)	0.63	(0.26, 1.53)
H	352/2071	1 (0.28)	3 (0.82)	0 (0)	10 (0.7)	13 (0.63)	0.45	(0.06, 3.46)
All	8257/21447	78 (0.94)	74 (1.18)	60 (0.57)	132 (2.86)	266 (1.24)	0.76	(0.59, 0.98)

### *Streptococcus agalactiae* isolated from heifers and cows

*Streptococcus agalactiae* was isolated in 7 heifer quarters (7 individuals) and 20 cow quarters (15 individuals). There was one case with *Streptococcus agalactiae* isolated from three quarters in the same individual and one case isolated from all four quarters. Separate generalized linear models were fit for the presence/absence of *Streptococcus agalactiae* and *Streptococcus agalactiae* at the quarter level to determine if an effect due to parity could be estimated while accounting for the nesting of quarters within individuals and herds. The coefficient estimates and 95% confidence intervals are presented in Table 3. There were no significant findings.

**Table 3:** Coefficient estimates and 95% confidence intervals for estimates from GLM models of Staphylococcus aureus and Streptococcus agalactiae with nested animal and herd random effects

**Parity**	***Staphylococcus aureus***	***Streptococcus agalactiae***
	**Coef. estimate (95% CI)**	**Coef. estimate (95% CI)**
1^st^ Lactation cows	1.31 (0.15, 11.62)	0.63 (0, 1.8E6)
2^nd^ Lactation cows	1.39 (0.14, 14.34)	0.53 (0, 1.8E7)
2^nd^ plus lactation cows	1.44 (0.21, 9.75)	1.18 (0,5.2E4)

All results are evaluated compared to the baseline category of heifer.

## Discussion

This study reported a much lower overall quarter prevalence of IMI in pregnant heifers, 34% (Table 1), compared to 70% to 97% previously reported [8,16,17]. Coagulase negative staphylococci (CNS) were isolated in 26% of heifer quarters and 11% of cow quarters, which correlates with other studies [8,11,16,18-21]. In one study, most CNS infections present during the peri-partum period were eliminated spontaneously, or with antibiotic treatment during early lactation [19].

The teat canal and skin of heifer calves can be colonized with environmental pathogens at a very young age [22]. Host adapted mastitis organisms live mainly within the udders of cows, creating a primary source of IMI within a dairy herd. The risk of calves becoming infected increases when reared on infected milk or colostrum [23], but there is no proof that bacteria can spread via the digestive tract to the udder parenchyme. It is more likely that pre-weaned calves suckling or licking each other or themselves, can enhance colonization of the teat skin. The latter may predispose them to IMI [17]. Heifers in contact with older cows prior to calving have an increased risk of clinical mastitis post calving [24], while biting flies can play a role in the transmission of *Staphylococcus aureus* between infected and uninfected heifers [25].

The presence of *Streptococcus agalactiae* in udders of heifers is presently unexplained. It has been noted [26] that both clinical and sub-clinical mastitis in the lactating cows, increase the risks for heifers to contract mastitis. One hypothesis is that IMI in heifers with *Streptococcus agalactiae* may occur when calves suckled each other after consuming contaminated milk. The presence of *Streptococcus agalactiae* in udder secretions of pregnant heifers should also be considered when managing positive herds. This means freshly calved heifers may be a source of infection and may be a risk for the re-introduction of *Streptococcus agalactiae* into the lactating herd.

Prevention of mastitis is based on reducing exposure to mastitogenic organisms and on enhancing the ability of the heifers’ immune systems. Early segregation of calves from dams, the use of individual pens pre-weaning, culling of calves that persist in suckling others, pasteurization of discarded milk and effective fly control should be practiced. Intramammary treatment of heifers prior to calving was found to be beneficial in reducing mastitis after calving and lowering the early lactation culling rate [8,21,22,25,27,28]. It has been suggested that blanket intramammary treatment with a lactating cow preparation, 14 days prior to the expected calving date in problem herds, should be used [11]. No significant difference in treatment efficacy was found based on intramammary treatments during the first, second or third trimester of heifer pregnancy [27,29]. Systemic antibiotics administered to heifers were not effective in reducing the prevalence of mastitis [30]. Knowledge is required in order to become more pro-active in heifer rearing to ensure udder health in heifers [31].

## Conclusions

The aims of the study were to determine the status of IMI in pregnant heifers and to determine the possible risk of heifer calves becoming infected with host adapted udder pathogens when co-housed pre-weaning and reared on fresh cows’ milk. The prevalence of coagulase negative staphylococci, *Staphylococcus aureus*, *Streptococcus agalactiae* and samples with no bacterial growth in heifers was 26%, 0.9%, 0.08% and 66%, respectively. The prevalence ratio between heifers and cows in seven of the eight herds was < 1 (Table 2), although this ratio was only significantly different from one in 4 of those seven herds. A single herd had a prevalence ratio significantly above 1, potentially indicating higher risk for heifer IMI compared to that of cows, although predicting the actual risk of IMI is complex and outside of the scope of this research. A simple comparison of *Staphylococcus aureus* IMI between parity classes resulted in significant differences (particularly when the outlying farm was omitted) but these effects did not hold after considering a model with nested random effects. The presence of *Streptococcus agalactiae* in the udder of pregnant heifers is uncommon, especially as IMI in adult cows of these herds were low. The importance of finding *Streptococcus agalactiae* IMI in heifers is that heifers can now not be ruled out as a source of new *Streptococcus agalactiae* outbreaks in dairy herds. Rearing calves on fresh milk may pose a risk to heifer udder health when co-housed, especially when waste milk is used. In order to prove conclusively however, that rearing co-housed calves on fresh milk increases the risk of IMI with *Staphylococcus aureus* and *Streptococcus agalactiae* in pregnant heifers, further research is needed that would include a control group of similarly managed heifer calves that are not fed fresh milk.

## Methods

### General information

The study was conducted in eight South African pasture based, commercial dairy herds. All known herds with a management system including seasonal calving and co-housed calf rearing, both uncommon practices in South Africa, were considered for study inclusion (9 herds). One herd was excluded as there were no heifers in the herd. Udder health was not a consideration in herd selection. All dry cows and pregnant heifers in all eight study herds were sampled. Farms were visited and farm managers and veterinarians were involved in obtaining relevant information regarding the parlour layout, routine management and calf rearing. Culling criteria on these farms were mainly based on reproductive failure, as very little was known of the udder health status. All parlour layouts were swingover with high milk lines, without automatic cluster removers. No strip cups or cowside tests were used. Hands of milkers and clusters were not disinfected prior to or during the milking process. Only post milking teat dip was applied. All lactating cows in each herd were dried off abruptly on the same day and cows received blanket dry-cow treatment. The dry-cow preparations used were either Curaclox DC (Cloxacillin 600 mg, Ampicillin 300 mg) from Norbrook (ARK-AH) P.O. BOX 10698, Centurion, 0046), or Cephudder (300 mg Cephapirin) from Intervet SA (MSD Animal Health) P.O. BOX 46, Isando, 1600). The milk withdrawal periods were 30 and 35 days, respectively. Dry cows were kept on dry-land camps where they calved down. The host adapted IMI found in udder secretions of dry cows close to calving could give an indication of IMI at calving. Pregnant heifers used in this study were removed within hours after birth as calves from their dams and were co-housed in groups of 5 to 10. They were bucket fed with fresh milk twice daily.

### Sampling schedules

Cows and late pregnant heifers were enrolled in the study and quarter secretion samples were taken during the dry period of the adult cows, at least 35 days after they had been dried off with blanket intramammary dry-cow remedies. Pregnant heifers were sampled on the same day as the cows in the respective herds. They were in their late pregnancies. Samples were taken aseptically in accordance to standard procedures, and were transported on ice to reach the milk laboratory (University of Pretoria, Faculty of Veterinary Science, Onderstepoort) for microbiological analysis, within 24 hours after sampling.

### Identification of bacteria

The udder secretion samples were plated out on plates containing Columbia Agar base with 5% defibrinated bovine blood (Quantum Biotechnologies (Pty) Ltd, Ferndale, South Africa). Test plates were incubated for 24 – 48 hours at 37°C ± 1°C. With each batch of Columbia Agar base plates made, control plates were evaluated for sterility. Isolated bacteria were identified in accordance with standard laboratory milk culture methodology, based on colony morphology, haemolysis, the catalase test, KOH test and Gram staining. Additional tests included a Strepkit (Latex agglutination test from Quantum Biotechnologies (Pty) Ltd, Ferndale, South Africa), Staphylase Test (Quantum Biotechnologies (Pty) Ltd, Ferndale, South Africa) and the API 20E kit (BioMerieux, P.O. Box 4328, Honeydew, 2040) [32,33].

### Statistical analysis

Data were captured on the Milk Sample Diagnostic (MSD) commercial software computer program (Aretsi SA) [34] developed from the Milk Laboratory. Statistical analysis was carried out using R v 2.15.1 [35]. Confidence intervals for the prevalence ratios were calculated using the formula provided in [36]. Fisher’s exact test was used to compare IMI counts between groups of different parity classes, statistical significance was taken to be p < 0.05. Generalized linear models were developed for the proportion of infected quarters to assess differences attributable to parity [37]. A binomial family with logit link function was used, with nested random effects for animal and herd. A positive quarter was defined by the presence of a pathogen. Parity was included as a categorical variable with four levels: heifer, 1^st^ lactation cows, 2^nd^ lactation cows, 2^rd^ plus lactation cows.

## Competing interests

The authors declare that they have no competing interests.

## Authors’ contributions

IMP drafted the manuscript and critically revised it, formatted and edited the manuscript and was involved in the data analysis. JK was involved in reviewing the technical aspects of the manuscript as well as assisting in data analysis, literature review and drafting of the manuscript. JCW and RB were involved in the sample analysis as well as data input. ML re-evaluated the data, provided statistical analysis and contributed to the writing of sections in the text pertaining to statistical analysis. All authors read and approve the final manuscript.
